# Clinical Features and Outcomes of Peripheral Vascular Disease Patients Receiving Red Blood Cell Transfusions

**DOI:** 10.7759/cureus.3682

**Published:** 2018-12-04

**Authors:** Aravinda Nanjundappa, Molly John, Stephanie Thompson, Frank H Annie, Sarah Embrey, Vallabh Karpe, Ali Farooq, Alfred Tager

**Affiliations:** 1 Cardiology, Charleston Area Medical Center, Charleston, USA; 2 Internal Medicine, Charleston Area Medical Center, Charleston, USA; 3 Miscellaneous, Charleston Area Medical Center, Charleston, USA; 4 Pharmacy, University of Charleston, Charleston, USA; 5 Emergency Medicine, Charleston Area Medical Center, Charleston, USA

**Keywords:** peripheral vascular disease, red blood cell transfusions, peripheral vascular disease, pvd

## Abstract

Background

Peripheral vascular disease (PVD) patients are commonly transfused with red blood cells (RBC) due to their inability to compensate for anemia and blood loss. Anemias, as well as allogeneic transfusions, have been demonstrated as independent risk factors for increased mortality and morbidity following cardiovascular procedures. The relationships between anemia, transfusion, and adverse outcomes in PVD patients remain unascertained and understudied.

Methods

A retrospective cohort study was conducted to determine mortality at 30-day, one-year, and three-year markers among 330 randomly selected PVD patients. The clinical features of patients receiving transfusions were examined, and the mortality rates were compared between patients who received an RBC transfusion and those who did not. Cox regression analysis was employed to identify independent variables predicting mortality.

Results

Transfusions were found to have increased mortality rates over non-transfused patients at 30 days (6.1% vs. 1.8%, *p* = 0.05), one year (21.8% vs 12.1%, *p* = 0.02), and three years (41.2% vs. 23.0%, *p* = 0.001). Using a multivariate regression model, it was determined that the transfusion itself was not a significant cause of this decrease in survival, while the propensity to transfuse was a predictor for both short (30 days, 36.73 [1.85-728.06], *p* = 0.04) and long-term mortality (one year (8.83 [2.62-29.77], *p* < 0.001; three years (7.07 [1.46-8.07], *p* <0.01). Anti-coagulation therapy using intravenous (IV) heparin and the chronic comorbidities of coronary artery disease and diabetes mellitus were also robust independent predictors of decreased survival.

Conclusion

This study was able to find an association between RBC transfusion and reduction in short-term (three months) and long-term (three years) survival. Those requiring IV heparin during the hospital stay were at an increased risk of requiring blood transfusion, and patients receiving IV heparin were also found to have a significant increase in mortality rates.

## Introduction

Peripheral vascular disease (PVD) presents as inadequate tissue perfusion due to atherosclerosis and can lead to impaired quality of life, chronic infections, limb ischemia, limb loss, stroke, and death. Furthermore, PVD patients often have significant comorbidities including coronary artery disease, diabetes mellitus, and chronic kidney disease, complicating their care and prognosis. Anemia resulting from acute blood loss or iron deficiency can exacerbate the imbalance between oxygen supply and demand in PVD patients. Thus, anemia has been identified as an independent risk factor for adverse outcomes in patients with acute coronary syndromes [1], in those receiving either percutaneous coronary intervention [2-3] or cardiac surgery [4]. The severity of preoperative anemia significantly predicted 30 days and five-year major adverse cardiac events in patients undergoing non-cardiac elective open vascular surgery. In patients with advanced PVD (Rutherford 12 category 4 or 5) and receiving percutaneous angioplasty, hemoglobin levels below 10.5 g/dl were associated with an increased risk of adverse outcomes defined by target lesion revascularization, limb amputation, or death (hazard ratio, 4.17 [1.56-11.16], *p* < 0.004) [5].

Due to baseline anemia or procedural-related decreases in the hemoglobin levels, patients undergoing peripheral vascular procedures commonly receive red blood cell (RBC) transfusions [6]. Despite how frequently patients receive blood, transfusion practices vary widely among hospitals and clinicians with this variation thought in large part due to the lack of evidence-based guidelines on appropriate transfusion thresholds [7]. Physicians often aggressively transfuse PVD patients due to their being a high-risk population and believed to tolerate anemia poorly. RBC transfusions have been shown in both medical and surgical patients to associate with an increased occurrence of infections, pneumonia, impaired pulmonary function, multiple organ failure, more extended intensive care unit and hospital lengths of stay, and short- and long-term mortality [8]. After adjustment for transfusion propensity and patient and procedural characteristics, RBC transfusion significantly (*p *< 0.005) predicted of mortality, sepsis, and pulmonary distress (prolonged ventilation, pneumonia, or unplanned intubation) in patients who underwent lower-extremity revascularization. PVD patients, especially those with ulcerations and non-healing wounds, have an increased risk of infection and may be especially vulnerable to transfusion-related infectious complications [9]. Little is known about the effects of RBC transfusion in the PVD patients, and the current transfusion practices in this patient population are not well described in the literature. Thus, the purpose of our study was to determine whether the benefits of blood transfusions in the PVD patients outweigh the inherent risks in regard to short-term and long-term mortality [10-11].

## Materials and methods

We retrospectively analyzed patients aged 18 years or older who underwent a peripheral vascular procedure for either the treatment or diagnosis of PVD between January 1, 2006, and December 31, 2007, at the Charleston Area Medical Center, Charleston WV. To be included in the study sample, patients had to receive blood typing/cross-matching. Patients undergoing open-heart procedures or thoracotomies during the index hospitalization in addition to a peripheral vascular intervention were excluded. Patients presenting with ruptured abdominal aorta aneurysms were excluded. Patient lists were developed by selecting patients who had ICD-9 (International classification of disease) diagnosis codes relating to PVD and received vascular interventions identified by ICD-9 procedure codes. All the diagnosis and procedure codes have been listed in Table 1. All aspects of the study were approved by the Institutional Review Board of the Charleston Area Medical Center. 

**Table 1:** ICD-9 diagnosis and procedure codes

ICD-9 Diagnosis	ICD-9 Procedure Codes
433.10, 433.11 Occlusion and stenosis of carotid arteries	00.61, 00.63 Percutaneous angioplasty or atherectomy of an extracranial vessel, insertion of carotid artery stents
440.xx Atherosclerosis	38.0x Incision of vessel, embolectomy, or thrombectomy
441.4 Abdominal aortic aneurysm	38.1x Endarterectomy of vessel
443.9 Peripheral vascular disease, not specified	38.4x Resection of the abdominal aorta with replacement
	39.22-29 Vascular bypass including aorta-subclavian-carotid bypass and aorta-iliac-femoral bypass
	39.5 Percutaneous transluminal angioplasty of non-coronary vessels
	39.42, 39.53 Revision of arteriovenous shunt, repair of the arteriovenous fistula
	39.7x Endovascular repair of a vessel (39.73 not included)
	39.79 Other endovascular procedures on other vessels
	39.90 Insertion of non-drug-eluting peripheral vessel stents
	88.48 Arteriography of femoral and other lower-extremity arteries

Electronic medical records were used to collect information on patient demographics (age, gender, race, body mass index), past medical history (coronary artery disease, hypertension, diabetes mellitus, hyperlipidemia), and tobacco use. Admission via the emergency department, medications at the time of admission, and the use of intravenous (IV) heparin therapy were obtained from chart review. Collected laboratory data included hemoglobin levels (baseline, nadir, and on the day of transfusion) and baseline estimated glomerular filtration rate (eGFR) as determined by the modification of diet in renal disease equation. Kidney insufficiency was defined as an eGFR of <60 ml/min/1.73 m^2^. Patients were classified as being anemic using baseline hemoglobin values and World Health Organization criteria for anemia. According to this definition, men with preprocedural hemoglobin <13 g/dL and women with preprocedural hemoglobin <12 g/dL were classified as anemic. To determine the impact of nadir hemoglobin on receiving RBC transfusion, a dichotomous variable was created using the nadir hemoglobin value of above or below median value for the sampled patients.

The primary outcome was all-cause mortality in patients receiving blood transfusions compared to those who did not. The Social Security Death Index was used to determine the mortality rates. The one-year mortality rate of patients with PVD who underwent a vascular intervention was estimated at 12.5% in the non-transfused group. A relative risk of 2.0, i.e. a 25% of one-year mortality rate in the transfused patients, was considered clinically significant. A sample size calculation was performed at a two-sided 5% significance level. The study was designed to have a statistical power of 80% to detect an additional 12.5% increase in mortality. Power analysis resulted in a total sample size of 330 patients, with 165 randomly selected patients in each transfusion cohort.

The patient demographics and clinical features of baseline, nadir, and day of transfusion hemoglobin, the number of units of RBC transfused, and vital sign measures of transfused patients were analyzed using descriptive statistics. Patient characteristics, comorbidities, and intervention procedure-related factors were compared between patients receiving blood transfusions and non-transfused patients, using Chi-square tests or Fisher’s exact tests for categorical data. Continuous variables are displayed as medians (interquartile ranges) and were compared with the nonparametric Mann-Whitney U test. Outcome data were examined by the Kaplan-Meier analysis and compared between transfused and non-transfused patients by log-rank test. Multivariate analysis of predictors of mortality was performed using time-dependent Cox proportional hazards regression with forward stepwise selection using alpha entry and exit criteria of *p*-value >0.10 and <0.15, respectively. Cox regression analysis for predictors of mortality was performed adjusting for age, gender, coronary artery disease, diabetes mellitus, hypertension, hyperlipidemia, renal insufficiency, tobacco use, IV heparin therapy, anemia at baseline, nadir anemia below median, carotid procedure, open procedure, endovascular procedure, admission status, amputation, and RBC transfusion. Hazard ratios are reported with a 95% confidence interval (CI). To account for the confounding effect between transfusions and clinical features, a propensity score for RBC transfusions was constructed via logistic regression analysis and included in the multivariate model. The propensity model’s discrimination was assessed by the goodness of fit with the Hosmer-Lemeshow statistic, and its predictive performance was assessed with the C statistic. IBM SPSS Version 19.0 was used for all statistical analysis. For all analyses, statistical significance was set as a *p*-value of ≤0.05.

## Results

Clinical features of transfused patients

From January 1, 2006, to December 31, 2007, 653 patients received both PVD intervention and blood typing/cross-matching at our institution. From this population, we randomly selected 165 patients who received at least one unit of RBC during their admission with an equal number of randomly selected patients who did not receive RBC transfusion. The overall median age at procedure was 71 years (interquartile range: 62-79) with age not differing between patients receiving and not receiving transfusion (*p* = 0.58, Table 1). Females constituted a greater proportion of patients in the transfusion cohort (57.0%) than the non-transfusion group (43.0%, *p* = 0.02). The majority of the study sample was Caucasian (96.1%), with racial distribution not differing between the transfusion cohorts (*p *= 0.36). The co-morbidities of coronary artery disease, diabetes mellitus, hypertension, and hyperlipidemia were equally prevalent in the two transfusion cohorts.

By contrast, kidney insufficiency was more common in patients receiving RBC transfusion (50.9%) versus those not receiving transfusion (34.5%, *p* = 0.004). Receiving IV heparin for anticoagulation therapy was associated with high rates of RBC transfusion (*p* = 0.001). Home medication use at the time of admission did not differ.

**Figure 1:**
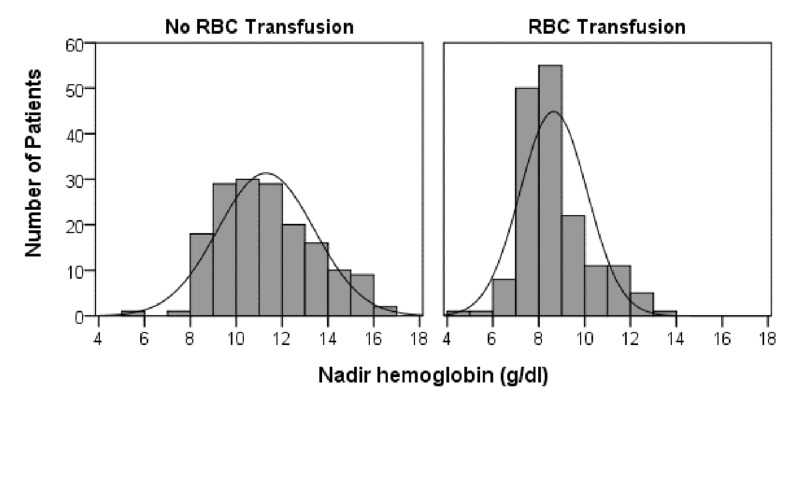
Histogram of nadir hemoglobin values The median nadir hemoglobin value for the 330 patients was 9.4 g/dl (interquartile range: 8.4-11.4). Patients receiving RBC transfusions had significantly lower nadir hemoglobin values (*p* < 0.001) and had a higher proportion of patients with nadir hemoglobin values below the median (*p* < 0.001). RBC: red blood cell

In the 165 patients receiving RBC transfusion, one RBC unit was transfused in 21.8% of patients, two units in 42.4%, and three or more units in 35.8% (median [interquartile range] = 2 [2 to 4 units]). The maximum number of RBC units transfused in an individual was 15. Hemoglobin levels obtained prior to the procedure were significantly lower in the transfused patients (11.80 g/dl [8.80-14.80] vs. 13.50 g/dl [10.50-16.50], *p *< 0.001), and as such, anemia at baseline was more frequent in patients receiving RBC transfusions (63.0%) versus patients without RBC transfusion (30.9%, *p *< 0.001). Transfused patients compared to not transfused individuals had significantly lower nadir hemoglobin values (8.50 g/dl [6.90-10.10] vs.11.10 g/dl [7.80-14.40], *p *< 0.001) and had a higher proportion of patients having nadir hemoglobin values below the median value of all patients (median value = 9.4g/dl, 76.4% vs. 20.0%, *p *< 0.001). Histograms of nadir hemoglobin values from both transfusion cohorts are shown in Figure 1. In patients receiving RBC, the median hemoglobin level on the day of transfusion was 8.70 g/dl (5.70-11.70).

**Figure 2:**
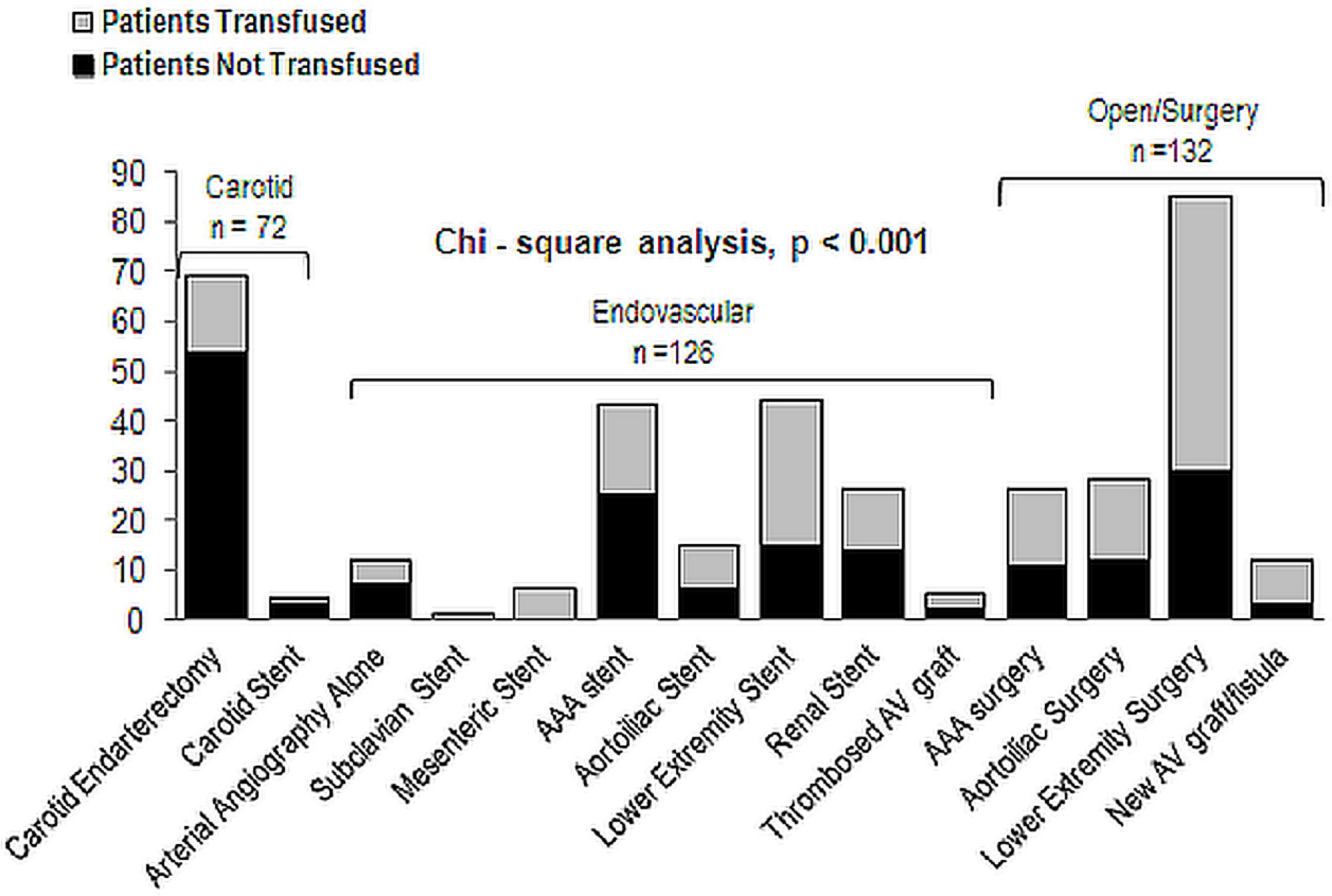
Variation across procedures in use of red blood cell transfusion for patients undergoing peripheral vascular disease diagnosis and intervention The rate of transfusion significantly differed between carotid, open surgical, and endovascular procedure types (Chi-square, *p* < 0.001).

The two most common PVD interventions in our study sample were carotid endarterectomy (69 patients) and lower-extremity surgery (85 patients). Figure 2 details the distribution of performed interventions and the rate of RBC transfusion within each procedure. Of the 330 examined patients, 132 individuals received open surgical procedures, 126 endovascular interventions, and 72 carotid procedures (either endarterectomy or stenting). Rates of RBC transfusions were significantly different between the three procedure categories (*p* < 0.001), with transfusions more frequent in open surgical procedures (84 of 132 patients, 63.6%), while transfusions were less frequent in carotid interventions (16 of 72 patients, 22.2%) Twelve patients received arterial angiography for the diagnosis of PVD in the absence of other responses, with five of them receiving transfusions during hospitalization. In individuals receiving endovascular interventions, the number of patients receiving RBC transfusion (65 patients, 52.8%) was nearly equal to that not obtaining transfusion (61 patients, 48.2%).

Mortality

**Figure 3:**
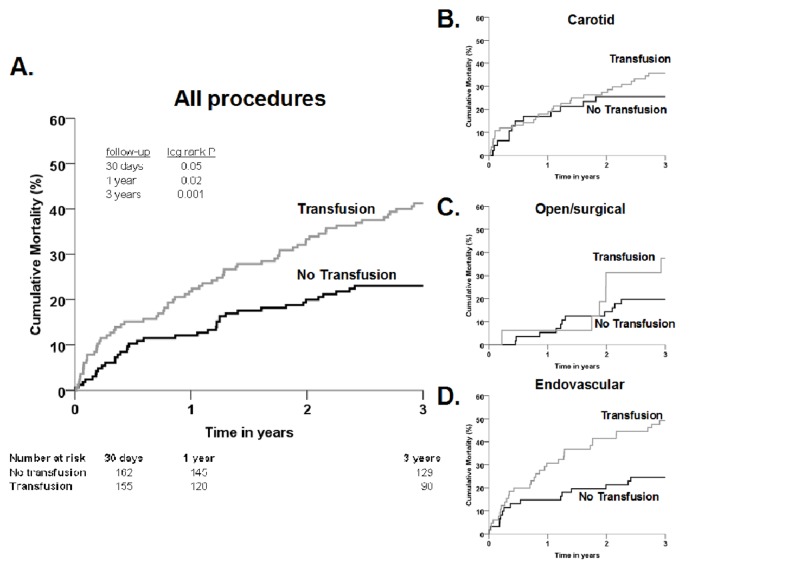
Kaplan-Meier estimates of mortality among patients who did and did not receive RBC transfusion Figure 3A: All procedures. Log-rank analysis demonstrated significantly increased mortality in transfused patients versus non-transfused patients at 30 days (*p* = 0.05), one year (*p* = 0.02), and three years (*p* = 0.001). Figure 3B: Carotid procedures; 72 patients. There was no difference in survival rates between transfused and non-transfused patients (30 days, no deaths; one year, *p* = 0.87; and three years, *p* = 0.16). Figure 3C: Open surgical procedures; 132 patients. There was no difference in survival rates between transfused and non-transfused patients (30 days, *p* = 0.22; one year, *p* = 0.83; and three years, *p* = 0.25). Figure 3D: Endovascular procedures; 126 patients. Mortality was significantly higher in patients receiving RBC transfusion at one year (*p* = 0.04) and three years (*p* = 0.006) but not 30 days (*p* = 0.46) post-procedure.

RBC transfusions were associated with decreased short- and long-term survival. A small percentage (3.9%) of the total sample population died within 30 days of their procedure with increased mortality occurring in the transfused cohort over those not-transfused (6.1% vs. 1.8%, *p* = 0.05). Additionally, log-rank analysis showed a significantly increased mortality in patients receiving transfusion versus those not receiving RBC transfusions at one year (21.8% vs 12.1%, *p* = 0.02) and three years (41.2% vs. 23.0%, *p* = 0.001, Figure 3A). All-cause mortality was examined in each of the procedure subtypes (carotid, open surgical, and endovascular). We found no difference at any examined period in mortality between the transfused and non-transfused patients receiving either carotid (*p* > 0.16 at each of the time points, Figure 3B) or open surgical procedures (*p* > 0.22, Figure 3C). Conversely, we found in the patients receiving endovascular procedures that mortality was significantly higher in patients receiving RBC transfusion at one year (*p* = 0.04) and three years (*p* = 0.006) post-procedure than the patients without transfusion (Figure 3D).

The propensity model for RBC transfusion included females (odds ratio [95% CI]: 1.95 [1.17 to 3.24], *p* = 0.01), non-carotid procedures (3.64 [1.79 to 7.39], *p* = 0.001), open surgical procedures (2.05 [1.16 to 3.62], *p* = 0.01), renal insufficiency (1.63 [0.94 to 2.81], *p* = 0.08), baseline anemia (2.80 [1.58 to 4.68], *p* = 0.001), and lower nadir hemoglobin levels (0.78 [0.73 to .83] for each 1.0 g/dl increase, *p* < 0.001). The C statistic of 0.82 from the model and the *p* value of 0.62 from the Hosmer-Lemeshow goodness of fit test suggest a good model fit.

Upon multivariate analysis, RBC transfusion failed to be included in the stepwise model and thus was not an independent predictor of mortality at 30 days, one year, and three years post-procedure (Table 1). The propensity to transfuse remained a significant predictor of mortality at 30 days (36.73 [1.85-728.06], *p* = 0.04), one year (8.83 [2.62-29.77], *p* < 0.001), and three years (7.07 [1.46-8.07], *p* < 0.01). Chronic comorbidities that significantly attributed to long-term mortality were coronary artery disease and diabetes mellitus. Additionally, at all three examined survival periods, IV heparin therapy significantly predicted decreased survival.

## Discussion

PVD is a systemic process, and the patients typically have multiple comorbidities, often including cardiovascular disease, diabetes, and/or chronic kidney disease. Previous studies on PVD-related procedures have found that the populations more likely to receive RBC transfusion included older individuals, women, and those with hypertension, diabetes, and renal insufficiency [12-13]. Poorer clinical outcomes in various patient populations have also been linked to anemia and RBC transfusions [14]. The significant findings that can be concluded from our study examining PVD-related endovascular and surgical procedures include: (1) those with anemia at presentation are more likely to receive transfusion; (2) patients administered with IV heparin were more likely to receive RBC transfusion and were also found to have significantly decreased survival; (3) those having the comorbidities of coronary artery disease and/or diabetes mellitus had increased rates of death regardless of transfusion status; and (4) while blood transfusions associated with increased short-term and long-term mortality when examined independently, in the presence of confounders including the risk of transfusion, RBC transfusion did not associate with increased mortality.

Transfusion of RBC is certainly beneficial for some select patients; however, there is demonstrable evidence of its association with adverse outcomes [14]. Blood transfusion has been found to be potentially thrombogenic and has also been known to suppress immune function. Transfusion-induced lung injury (TRALI) is associated with increased mortality [15]. Blood transfusions in PVD patients may induce further cardiovascular and kidney damage [12]. However, it remains difficult to determine if adverse outcomes are due to patient characteristics and comorbidities (including anemia) that predispose patients to receive transfusions or the transfusion itself.

In the presence of confounders, blood transfusions are associated with increased mortality following hospitalization for acute coronary syndrome [16], percutaneous coronary intervention [17], and open vascular surgeries [13]. However, in our current study, RBC transfusion did not associate with increased short- or long-term mortality following PAD-related procedures in the presence of confounders. Our findings differed from those of O’Keeffe et al. who found that after adjustment for transfusion propensity and patient and procedural characteristics, RBC transfusion significantly predicted of mortality, sepsis, and pulmonary distress in patients who underwent lower-extremity vascular surgery [8]. Additionally, post-procedure transfusion highly correlated with in-hospital mortality and morbidity (myocardial infarction, stroke, and acute renal failure) in patients receiving lower-extremity endovascular procedures following adjustment of confounders using the propensity scores for transfusion [12]. However, both the studies of O’Keeffe et al. [8] and Henke et al. [12] used large registries (the American College of Surgeons National Surgical Quality Improvement Project [ACS NSQIP] and the Blue Cross/Blue Shield of Michigan Cardiovascular Consortium, respectively) in their data analysis, while our sample size was smaller and at a single institution. Furthermore, many of the studies examining the relationship between RBC transfusion and adverse events examined mortality only in-hospital [12,17] or at 30 days [8,13,16]. In one study though, RBC transfusion was found to associate with the composite endpoint of death and myocardial infarction up to two years post vascular surgical procedure [18].

The Hgb levels at which the benefits of transfusion outweigh the risks and financial cost and the patient groups that benefit the most from RBC transfusions are currently unknown. Restrictive transfusion guidelines and judicious use of blood transfusions may be of benefit in PVD patients, with restrictive transfusion strategies demonstrating reduced transfusion rates in the absence of worse patient outcomes [19-20]. Furthermore, the association of coronary artery disease and diabetes mellitus with increased mortality highlights the importance of continued evidence-based clinical management of PVD patients’ often numerous comorbidities. 

## Conclusions

In the presence of confounders, including the likelihood of transfusion, RBC transfusion did not associate with increased mortality following endovascular and open, surgical vascular procedures. We suggest in patients with PVD, that other factors, besides RBC transfusion, such as the comorbidities of coronary artery disease and/or diabetes mellitus, are more closely associated with all-cause death than the transfusion status.

## References

[1] Meneveau N, Schiele F, Seronde MF (2009). Anemia for risk assessment of patients with acute coronary syndromes. Am J Cardiol.

[2] McKechnie RS, Smith D, Montoye C (2004). Prognostic implication of anemia on in-hospital outcomes after percutaneous coronary intervention. Circulation.

[3] Nikolsky E, Aymong ED, Halkin A (2004). Impact of anemia in patients with acute myocardial infarction undergoing primary percutaneous coronary intervention: analysis from the controlled abciximab and device investigation to lower late angioplasty complications (CADILLAC) trial. J Am Coll Cardiol.

[4] Carson JL, Duff A, Poses RM (1996). Effect of anaemia and cardiovascular disease on surgical mortality and morbidity. Lancet.

[5] Toor IS, Jaumdally RJ, Moss MS, Babu SB (2009 ). Preprocedural hemoglobin predicts outcome in peripheral vascular disease patients undergoing percutaneous transluminal angioplasty. J Vasc Surg.

[6] Glance LG, Dick AW, Mukamel DB (2011). Association between intraoperative blood transfusion and mortality and morbidity in patients undergoing noncardiac surgery. Anesthesiology.

[7] Retter A, Wyncoll D, Pearse R (2013). Guidelines on the management of anaemia and red cell transfusion in adult critically ill patients. Br J Haematol.

[8] O'keeffe SD, Davenport DL, Minion DJ (2010). Blood transfusion is associated with increased morbidity and mortality after lower extremity revascularization. J Vasc Surg.

[9] Shaw RE, Johnson CK, Ferrari G (2013). Balancing the benefits and risks of blood transfusions in patients undergoing cardiac surgery: a propensity-matched analysis. Interact Cardiovasc Thorac Surg.

[10] Dunkelgrun M, Hoeks SE, Welten GM (2008). Anemia as an independent predictor of perioperative and long-term cardiovascular outcome in patients scheduled for elective vascular surgery. Am J Cardiol.

[11] Rogers MA, Blumberg N, Saint S, Langa KM, Nallamothu BK (2009). Hospital variation in transfusion and infection after cardiac surgery: a cohort study. BMC Medicine.

[12] Henke PK, Park YJ, Hans S (2016). The association of peri-procedural blood transfusion with morbidity and mortality in patients undergoing percutaneous lower extremity vascular interventions. PloS one.

[13] Obi AT, Park YJ, Bove P (2015). The association of perioperative transfusion with 30-day morbidity and mortality in patients undergoing major vascular surgery. J Vasc Surg.

[14] Salpeter SR, Buckley JS, Chatterjee S (2014). Impact of more restrictive blood transfusion strategies on clinical outcomes: a meta-analysis and systematic review. Am J Med.

[15] Toy P, Popovsky MA, Abraham E (2005). Transfusion-related acute lung injury: definition and review. Crit Care Med.

[16] Rao SV, Jollis JG, Harrington RA (2004). Relationship of blood transfusion and clinical outcomes in patients with acute coronary syndromes. JAMA.

[17] Marik PE, Corwin HL (2008). Acute lung injury following blood transfusion: expanding the definition. Crit Care Med.

[18] Jones AR, McGhan G, Deaver J (2017). Packed red blood cell transfusion in older adults: a systematic review. J Gerontol Nurs.

[19] Kooby DA, Stockman J, Ben-Porat L (2003). Influence of transfusions on perioperative and long-term outcome in patients following hepatic resection for colorectal metastases. Ann Surg.

[20] Jy W, Ricci M, Shariatmadar S, Gomez‐Marin O, Horstman LH, Ahn YS (2011). Microparticles in stored red blood cells as potential mediators of transfusion complications. Transfusion.

